# Can Zinc Supplementation Attenuate High Fat Diet-Induced Non-Alcoholic Fatty Liver Disease?

**DOI:** 10.3390/ijms24021763

**Published:** 2023-01-16

**Authors:** Oluwanifemi Esther Bolatimi, Kimberly Z. Head, Jianzhu Luo, Tyler C. Gripshover, Qian Lin, Ngozi V. Adiele, Walter H. Watson, Caitlin Wilkerson, Lu Cai, Matthew C. Cave, Jamie L. Young

**Affiliations:** 1 Department of Pharmacology and Toxicology, School of Medicine, University of Louisville, Louisville, KY 40202, USA; 2 Division of Gastroenterology, Hepatology and Nutrition, Department of Medicine, School of Medicine, University of Louisville, Louisville, KY 40202, USA; 3 Hepatobiology & Toxicology COBRE, School of Medicine, University of Louisville, Louisville, KY 40202, USA; 4 Superfund Research Program, University of Louisville, Louisville, KY 40202, USA; 5 Pediatric Research Institute, Department of Pediatrics, School of Medicine, University of Louisville, Louisville, KY 40202, USA; 6 The Center for Integrative Environmental Health Sciences, University of Louisville, Louisville, KY 40202, USA; 7 Alcohol Research Center, University of Louisville, Louisville, KY 40202, USA; 8 Department of Biochemistry and Molecular Genetics, School of Medicine, University of Louisville, Louisville, KY 40202, USA

**Keywords:** non-alcoholic fatty liver disease (NAFLD), high fat diet, liver injury, essential micronutrient, zinc supplementation, therapeutic agent

## Abstract

The pathogenesis of non-alcoholic fatty liver disease (NAFLD), the most prevalent chronic liver disease, is associated with zinc deficiency. Previous studies show zinc supplementation improves steatosis and glucose metabolism, but its therapeutic effects in patients with established NAFLD remain unclear. We developed an in vivo model to characterize the effects of zinc supplementation on high-fat diet (HFD) induced NAFLD and hypothesized that the established NAFLD would be attenuated by zinc supplementation. Male C57BL/6J mice were fed a control diet or HFD for 12 weeks. Mice were then further grouped into normal and zinc-supplemented diets for 8 additional weeks. Body composition and glucose tolerance were determined before and after zinc supplementation. At euthanasia, plasma and liver tissue were collected for characterization and downstream analysis. As expected, 12 weeks of HFD resulted in reduced glucose clearance and altered body composition. Eight weeks of subsequent zinc supplementation did not alter glucose handling, plasma transaminases, steatosis, or hepatic gene expression. Results from our model suggest 8-week zinc supplementation cannot reverse established NAFLD. The HFD may have caused NAFLD disease progression beyond rescue by an 8-week period of zinc supplementation. Future studies will address these limitations and provide insights into zinc as a therapeutic agent for established NAFLD.

## 1. Introduction

The global prevalence of obesity is paralleled by a corresponding increase in the severity and prevalence of various metabolic diseases, including non-alcoholic fatty liver disease (NAFLD). NAFLD represents a spectrum of diseases ranging from simple hepatic steatosis to more severe steatohepatitis in the absence of alcohol consumption [1]. Presently, NAFLD has surpassed alcohol-associated liver disease as the most common chronic liver disease, affecting up to 25% of the global adult population [2]. By the year 2030, the NAFLD population is projected to increase to 100.9 million cases, a 21% increase since 2015 [3]. With a wide range of etiologies, NAFLD is commonly attributed to excessive consumption of a high-calorie diet with poor nutritional content [2]. NAFLD, characterized by the accumulation of triglycerides in the liver, is regarded as the hepatic manifestation of an array of multifactorial events, such as metabolic syndrome, obesity, and diabetes [1,4,5,6].

Non-alcoholic fatty liver has become a major health concern as there are no overt symptoms of the disease, and, left untreated, it can progress into steatohepatitis (NASH), cirrhosis, or hepatocellular carcinoma [7]. The later stages of disease development require liver transplantation or lead to sudden and severe liver failure resulting in death [8]. Aside from these hepatic outcomes, NAFLD has associated non-liver consequences such as cardiovascular disease [3] and kidney dysfunction [9]. Upon the reduction of steatosis, liver injury is reversible. The current management of NAFLD consists of lifestyle changes, such as diet restriction and increased physical activity, but there is still a lack of effective therapeutics approved by the Food and Drug Administration for the treatment of NAFLD [3]. This necessitates a critical search for effective and appropriate therapeutic agents for the treatment of NAFLD.

The pathogenesis of NAFLD still remains unclear but is associated with mechanisms involving oxidative stress, insulin resistance, dyslipidemia, and deficiencies in micronutrients [10,11]. Deficiency in the micronutrient zinc, which affects 17.3% of the global population due to inadequate zinc intake [12], is implicated in playing a crucial role in the pathogenesis of NAFLD. Zinc is the second most concentrated trace mineral in the body [13]. This vital trace element contributes to a diverse range of biological processes serving structural, catalytic, signaling, and regulatory functions. Some of these include a role in energy metabolism, anti-inflammation, and antioxidative pathways [11,14]. Dysregulation in these processes is a risk factor and/or may increase susceptibility to the development of NAFLD. Zinc deficiency is most prevalent in developing and impoverished nations, but it is also increased in vegan and vegetarian populations, as well as in people with disorders associated with reduced zinc absorption and the elderly [15,16,17]. Clinical studies have demonstrated low serum levels of zinc in chronic hepatitis, cirrhosis, and liver cancer patients. Zinc levels are also correlated with disease severity and outcome. In particular, reduced serum zinc acts as an independent risk factor for significant hepatic fibrosis in NAFLD patients [18].

Zinc affects enzymes required for normal hepatic identity, function, and lipid metabolism [19]. Zinc has been shown to reduce hepatic lipid accumulation by preventing lipogenesis and stimulating lipolysis through autophagy-mediated lipophagy [20]. Hepatic zinc deficiency alters liver function and energy metabolic profiles, promoting hepatic lipid accumulation [21] and the development of NAFLD. Studies investigating zinc supplementation consumed concurrently with a high fat diet (HFD) show reduced hepatic steatosis and improved glucose metabolism [22]. Additionally, zinc supplementation has been shown to be protective in lowering serum glucose in rodent models [10]. While studies addressing the role of zinc supplementation prior to the development of NAFLD have been conducted, the therapeutic effects of dietary zinc supplementation following the establishment of NAFLD remain to be determined. 

Therefore, the objective of this study was to develop an in vivo model exploring dietary zinc supplementation as a potential therapeutic agent after the establishment of high-fat diet-induced NAFLD. We explored the effects of zinc supplementation on glucose metabolism, hepatic lipid accumulation, hepatic injury, and gene expression of hepatic enzymes, of which zinc is a major structural component.

## 2. Methods and Materials

### 2.1. Animals Model and Diets

**Figure 1:**
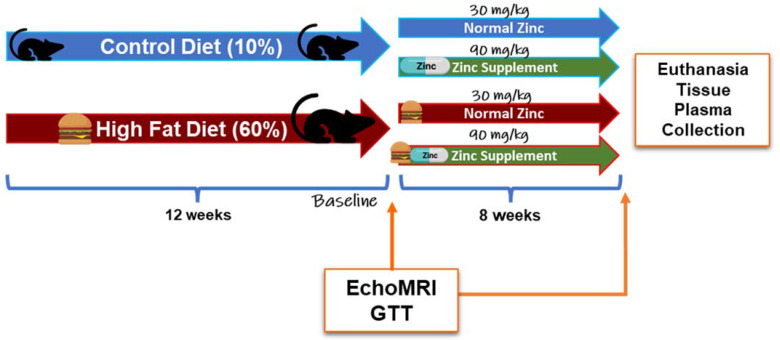
**Exposure paradigm.** 9-week-old male C57BL/6J mice were fed control (n = 30, 10% fat-kcal) or HFD (n = 30, 60% fat-kcal) for 12 weeks, after which they were further subdivided into normal zinc (n = 15, 30 mg zinc/4057 kcal) or zinc supplemented (n = 15, 90 mg zinc/4057 kcal) diet groups for an additional 8 weeks. Prior to zinc supplementation and animal sacrifice, IPGTTs were conducted, and body composition determined by Echo MRI. IPGTT = intraperitoneal glucose tolerance test.

Animal use, protocols, and procedures were approved by the University of Louisville Institutional Animal Care and Use Committee (OLAW/PHS Assurance No. A3586-01; USDA Registration No. 61-R-001-01). The University of Louisville is an Association for the Assessment and Accreditation of Laboratory Animal Care, International (AAALAC) accredited institution. Male C57BL/6J mice (8-weeks-old; n = 60) were purchased from Jackson Laboratory (Bar Harbor, ME, USA). Mice were housed in a temperature- and light-controlled room with a 12 h light/dark cycle. Mice were allowed to acclimate after the facility transfer for one week and were fed autoclaved standard laboratory rodent chow. After acclimation, mice were switched to purified diets to minimize the influence of metal contamination found in standard chow on experimental outcomes [23,24]. Specifically, mice were either fed a control diet (CD, 10% kcal fat; Research Diets D14020202, New Brunswick, NJ, USA) or a HFD (60% kcal fat; Research Diets D14020205, New Brunswick, NJ, USA) for 12 weeks. After 12 weeks, mice were further grouped into diets containing either 30 mg or 90 mg zinc/4057 kcal, representing the normal zinc (NZ) and added zinc (ZS) diets, respectively. (CD + Zn—Research Diets D14020203; HFD + Zn—Research Diets D14020206, New Brunswick, NJ, USA) for an additional 8 weeks [Figure 1]. Food and water were provided ad libitum. Body weights and food consumption were recorded weekly. Intraperitoneal glucose tolerance tests (IPGTT) were performed at weeks 12 and 19. Prior to euthanasia, the body composition of the animals was determined by quantitative magnetic resonance imaging using an EchoMRI-500 Body Composition Analyzer (Echo Medical Systems, Houston, TX, USA). At the end of 20 weeks, mice were anesthetized (ketamine/xylazine, 120/16 mg/kg body weight) via i.p. injection, followed by blood collection from the inferior vena cava and subsequent euthanasia via exsanguination. Blood samples were centrifuged, and plasma was stored at −80 °C for further biochemical analysis. Liver weight was recorded for each mouse, and portions of liver tissue were snap-frozen in liquid nitrogen, processed for RNA isolation, fixed in 10% neutral buffered formalin for histology, and used for metal analysis. The liver weight to tibia length ratio (liver weight in grams divided by tibia length in millimeters) was used as an index of liver size changes. Mice were fasted overnight prior to sacrifice.

### 2.2. Glucose Tolerance Test

IPGTT was performed at week 12, before zinc supplementation, and at week 19, one week prior to the end of the study. Mice were fasted for 6 h, weighed, and injected with glucose (2 mg glucose/g body weight, sterile saline, i.p.) [25,26]. Blood glucose levels were measured at 0, +15, +30, +60, and +120 min post-injection with a hand-held glucometer (Contour Next EZ, Parsippany, NJ, USA) using 1 µL of blood via tail prick. A time course of absolute blood glucose measurement and the area under the curve (AUC) were determined for each animal.

### 2.3. Blood Chemistry Analysis

Plasma alanine transaminase (ALT), aspartate transaminase (AST), triglycerides, cholesterol, glucose, high-density lipoprotein (HDL), low-density lipoprotein (LDL), and very low-density lipoprotein (VLDL) levels were measured using Lipid Panel Plus disks (catalog:400-0030; Abaxis Inc.; Union City, CA, USA) with the Piccolo Xpress Chemistry Analyzer (Abbott; Abbott Park, IL, USA).

### 2.4. Measurement of Hepatic Triglyceride and Cholesterol Content

Liver tissues were washed in 50 mM NaCl and homogenized in 1 mL 50 mM NaCl with 0.5 mm glass silica beads and beaded for 30 s. Hepatic lipids were extracted using an aqueous solution of chloroform and methanol in a 2:1 ratio based on the Bligh and Dyer method (Bligh and Dyer, 1959). Triglyceride and cholesterol standards (catalog: T7531-STD, C7509-STD; Point Scientific; Canton, MI) were utilized to generate a standard curve to quantify the extracted lipids. Extracted triglycerides and cholesterol were colorimetrically quantified using a microplate absorbance reader (BioTek Gen 5; Winooski, VT, USA). Reagents used in the assay: Infinity Triglycerides Reagent (TR22421, ThermoFisher Scientific, Middletown, VA, USA) and Infinity Cholesterol Reagent (TR13421, ThermoFisher Scientific, Middletown, VA, USA).

### 2.5. Liver Histology

Liver tissues were fixed in 10% neutral buffered formalin for at least 24 h and moved to 75% ethanol until tissue processing prior to being embedded in paraffin. Paraffin embedded tissues were sectioned at 5 µm with Leica Biosystem’s Histocore Autocut Automated Rotary Microtome (Leica Biosystem; Deer Park, IL, USA). Tissue sections were deparaffinized with a citrisolv hybrid, rehydrated with graded ethanol washes, and stained with either hematoxylin and eosin (H&E) to assess the overall hepatic structure or Masson Trichrome to assess the collagen deposition as an indicator for fibrosis using the Epredia™ Gemini™ AS Automated Slide Stainer (Fisher Scientific, Pittsburgh, PA, USA). Steatosis was scored as a percent of liver cells in a 10× field containing fat (<25% = 1+; <50% = 2+; <75 = 3+; >75% = 4+) (Nanji et al. 1989). For each animal, ten 10× fields were scored. 10× and 20× field Masson Trichrome images were taken, and five 10× and five 20× images were quantified for collagen deposition using ImageJ (v1.53k) software (National Institute of Health; Bethesda, MD, USA). Images were captured on cellSens Standard XV image processing software using the Olympus DP74 digital camera and Olympus BX43 microscope (Olympus America, Breinigsville, PA, USA).

### 2.6. Real-Time qPCR

Liver tissues were homogenized using 1 mL of chilled RNA-STAT 60^TM^ (catalog: CS502; Tel-test Inc.; Friendswood, TX) per 50–100 mg of tissue in a 2 mL screwcap tube containing 0.5 mm glass silica beads, followed by homogenization for 30 s using a mini-beadbeater (Sigma Aldrich, St. Louis, MO, USA). Total RNA was then extracted, precipitated, and washed following the RNA-STAT 60 reagent protocol for the isolation of total RNA, DNA, and protein by AMSBIO. RNA quantity and purity were assessed with Nanodrop One^C^ (Thermo Scientific, catalog: 701-058112; Madison, MI, USA). cDNA was reverse transcribed from 3 µg total RNA to yield 60 µL using the single step cDNA synthesis reagent QScript (Quantabio; Catalog: 95048-500), following the manufacturer’s protocol. qRT-PCR was performed on a CFX384 Touch Real-Time PCR Thermal Cycler (Bio-Rad, Hercules, CA) using iTaq Universal Probe Supermix (catalog:1725134; Biorad, Hercules, CA, USA) according to the manufacturer’s protocol. Primer sequences from TaqMan Gene Expression Assays (ThermoFisher Scientific) were as follows: hepatocyte nuclear factor 4 alpha (Hnf4α); (Mm01247712_m1), tumor necrosis factor alpha (TNFα); (Mm00443258_m1), peroxisome proliferator-activated receptor alpha (Pparα); (Mm00440939_m1), adiponectin (Adipoq); (Mm04933656_m1) and apolipoprotein B (Apob); (Mm01545150_m1). Levels of mRNA expression were calculated using the 2-ΔΔCt method. Fold induction was calculated and normalized relative to the amount of Glyceraldehyde-3-Phosphate Dehydrogenase (GAPDH) mRNA (catalog:4352339E, ThermoFisher Scientific; Madison, MI, USA), and expression levels of mice fed the control diet with no zinc supplementation, which were set to 1.

### 2.7. Metal Analysis

Each liver sample was weighed and digested in 600 µL of 70% concentrated trace metal grade nitric acid (Fisher Scientific) in a 65 °C shaking incubator for 4 h. After digestion, samples were cooled to room temperature and filtered using a 100 µm filter to remove undigested debris. Furthermore, 8.2 mL of Milli-Q deionized water was added to each sample, bringing the final concentration of nitric acid to 5%. Metal levels were assessed using an Agilent 7800 inductively coupled plasma mass spectrometry (ICP-MS) equipped with an Agilent SPS 4 autosampler (Agilent Technologies, Inc., Santa Clara, CA, USA) for sample injection. During sample injection, internal standards including Bi, In, Li, Sc, Tb, and Y (Inorganic Ventures) were mixed with each sample for drift correction and accuracy improvement. Each sample was analyzed three times, and metal levels were calculated and presented as µg/g wet tissue. Anything less than the intercept concentration was considered non-detectable.

### 2.8. Statistics

Statistical analyses were conducted using GraphPad Prism (version 9.2.0) for Windows (GraphPad Software Inc.; La Jolla, CA, USA). Data was expressed as mean ± SD. Two group comparisons were performed using an unpaired *t*-test. Multiple group data was compared using the Two-Way Analysis of Variance (ANOVA) followed by Bonferroni’s post-hoc test to correct for multiple hypothesis testing. Statistical significance was set at an alpha level of 0.05 (*p* ≤ 0.05).

## 3. Results

### 3.1. Body Weight

**Figure 2:**
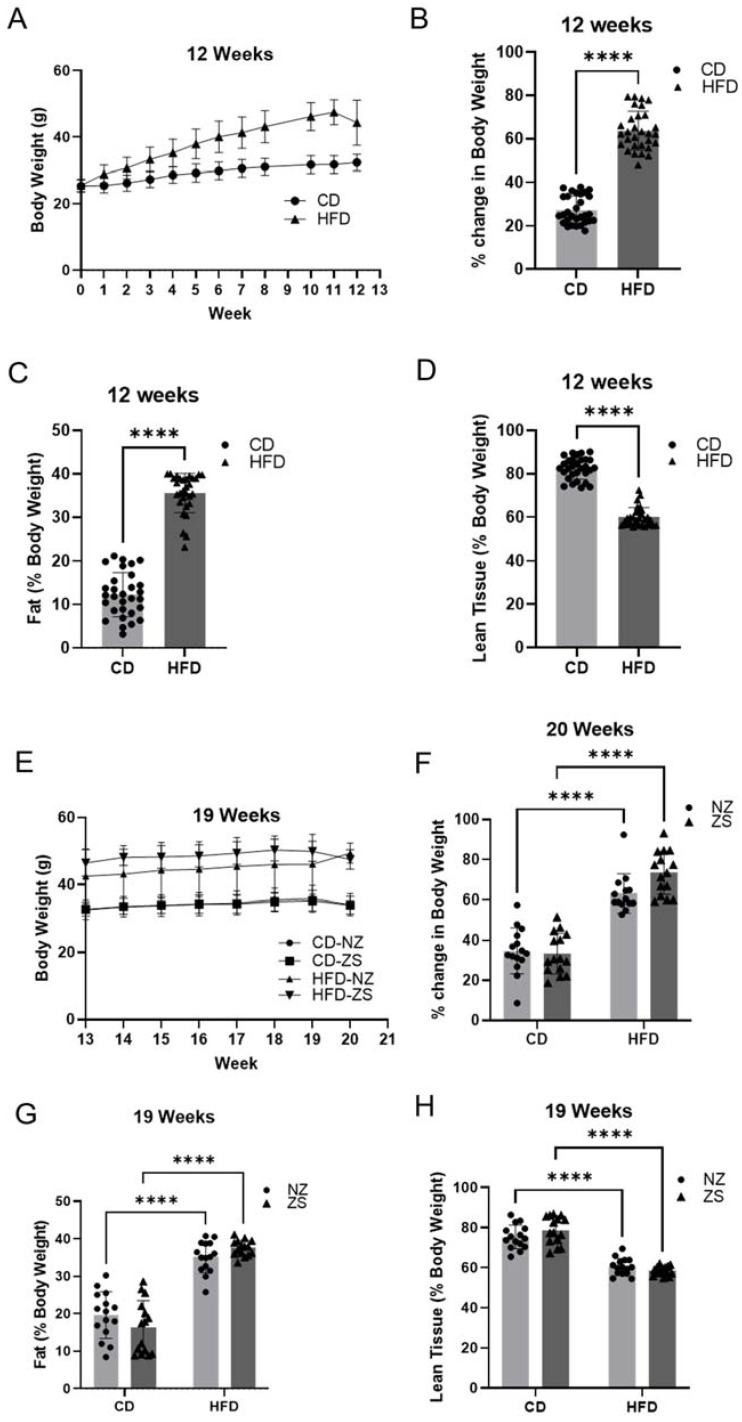
**Effects of HFD and zinc supplementation on body weight and composition.** 12 weeks of HFD resulted in significant body weight gain and altered body composition which zinc supplementation could not ameliorate. Body weights were measured weekly for duration of study and body composition determined by Echo MRI scan at weeks 12 and 19. Panels **A**–**D** show outcomes for weeks 1–12 (+/− HFD). Panels **E**–**H** show outcomes for weeks 13–20 (+/− HFD and +/− zinc supplementation). Data are reported as mean ± SD (n = 30 mice/group, **A**–**D**; n = 14–15 mice/group, **E**–**H**) with significance set to 0.05. **** *p* < 0.0001. NZ = normal zinc, ZS = zinc supplement.

Body weights were recorded weekly during the 20-week study period. All groups experienced weight gain, with HFD-fed mice gaining significantly more weight compared with CD-fed mice (*p* < 0.0001) (Figure 2A,B,E,F). Zinc supplementation had no significant effect on the mean percent body weight gain in both the HFD and CD zinc-supplemented mice (Figure 2E). As expected, HFD-fed mice experienced a significant increase in percent body fat (*p* < 0.0001) and a contrasting decrease in percent lean tissue (*p* < 0.0001) compared with CD-fed mice (Figure 2B,C,G,H). Similar to the effects on body weight, zinc-supplemented mice did not show significant differences in percent body fat or percent lean tissue compared with normal zinc-fed mice. The observed data suggest the HFD was successful in inducing obesity in HFD-fed mice.

### 3.2. Dietary and Hepatic Zinc Levels

**Figure 3:**
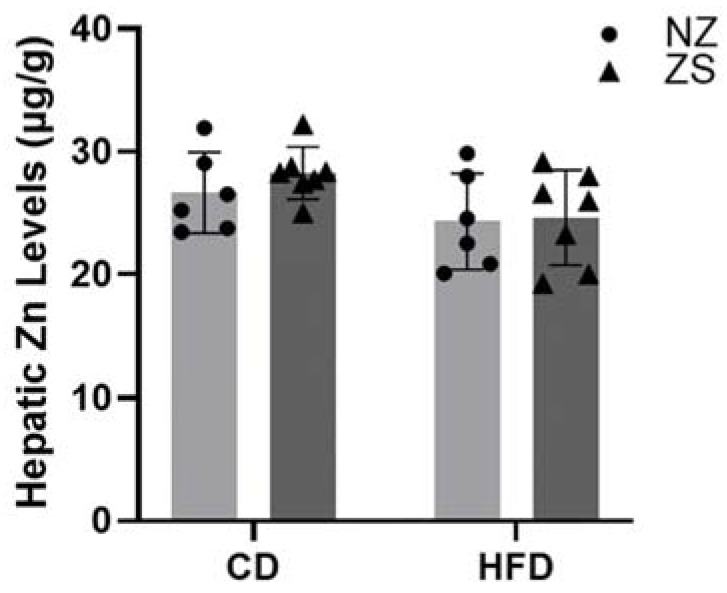
**Hepatic zinc levels.** Hepatic zinc content was measured by ICP-MS. Data are reported as mean ± SD (n = 14) significance set to 0.05. NZ = normal zinc, ZS = zinc supplement.

Mice were administered zinc in their food; therefore, we measured zinc levels in the diet and liver tissues. As expected, diets supplemented with zinc had three times the amount of zinc compared with levels in the normal zinc diets. CD with and without zinc supplement had 90.3 µg zinc/g of diet and 30.3 µg zinc/g of diet, respectively, while the HFD with and without zinc supplement had 124.0 µg zinc/g of diet and 40.0 µg zinc/g of diet, respectively. No significant differences were observed in hepatic zinc levels across groups (Figure 3). Zinc supplementation did not alter zinc accumulation in HFD, nor CD zinc supplemented mice compared with normal diet fed mice.

### 3.3. IPGTT

**Figure 4:**
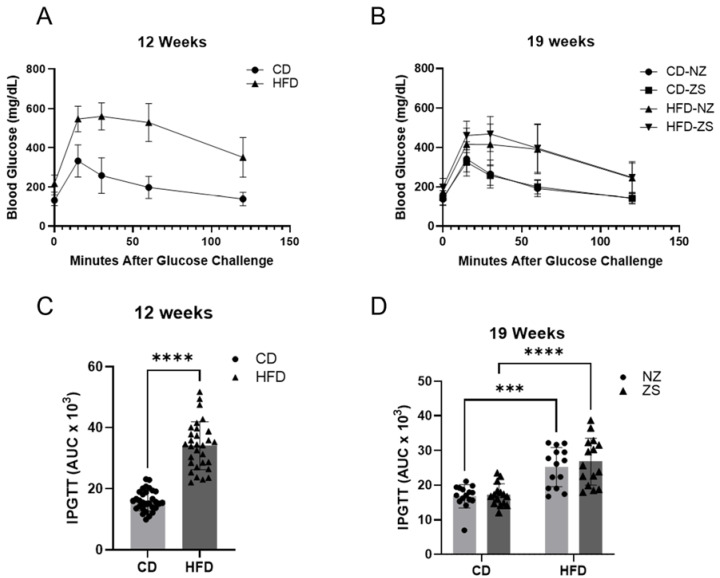
**Effects of HFD and zinc supplementation on glucose tolerance.** HFD induced increase in glucose tolerance was unaffected by zinc supplementation. Blood glucose levels in CD and HFD groups after IPGTT, performed (**A**) before zinc supplementation and (**B**) one week prior to sacrifice. (**C**,**D**) Integrated AUC was calculated showing higher changes in blood glucose levels in HFD groups. Data are reported as mean ± SD (n = 30 mice/group, **A**,**C**; n = 14–15 mice/group, **B**,**D**) with significance set to 0.05. *** *p* < 0.0004, **** *p* < 0.0001. CD = control diet, HFD = high fat diet, NZ = normal zinc, ZS = zinc supplement, IPGTT = intraperitoneal glucose tolerance test.

To assess glucose intolerance, often associated with NAFLD, glucose tolerance tests were performed at weeks 12 and 19. At week 12, HFD-fed mice had significantly increased blood glucose and AUC compared with CD-fed mice (Figure 4A,C) (*p* < 0.0001). After 7 weeks of subsequent zinc supplementation, HFD-fed mice still showed reduced glucose clearance and increased glucose AUC. However, zinc supplementation did not result in a significant difference in blood glucose and AUC between the supplemented and normal zinc groups. Similar observations were made in CD-fed mice. In summary, HFD was associated with reduced glucose clearance, of which zinc supplementation had no impact.

### 3.4. Plasma Lipids

Plasma total cholesterol, triglycerides, high-density lipoproteins (HDL), low-density lipoproteins (LDL), and very low-density lipoproteins (vLDL) levels were measured in all animal groups (Table 1). Overall, plasma cholesterol and HDL levels were increased due to HFD feeding, while triglyceride and vLDL levels decreased. Mean lipid levels were not significantly different between HFD zinc supplement and normal zinc-fed mice. Similarly, no significant differences were observed in CD zinc-supplemented compared with normal zinc-fed mice.

### 3.5. Hepatic Injury and NAFLD

**Figure 5:**
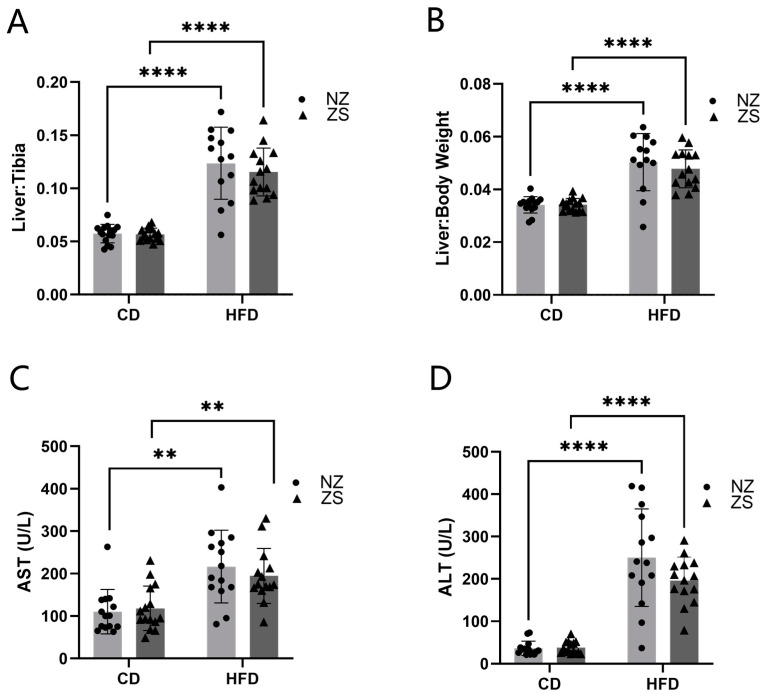
**Effects of HFD and zinc supplementation on liver injury.** HFD-induced liver injury was not reduced by zinc supplement. (**A**,**B**) Ratio of liver to tibia and body weight, measures of hepatomegaly. (**C**) Plasma aspartate aminotransferase (AST) and (**D**) alanine aminotransferase (ALT) activities were increased in HFD fed groups. Data are reported as mean ± SD (n = 14–15 mice/group) with significance set to 0.05. ** *p* < 0.05, **** *p* < 0.0001. CD = control diet, HFD = high fat diet, NZ = normal zinc, ZS = zinc supplement.

Liver-to-tibia and liver-to-body weight ratios were calculated as markers for hepatomegaly. HFD solely resulted in enlarged livers compared with CD (Figure 5A,B). No significant differences in either liver-to-tibia or liver-to-body weight ratios between zinc-supplemented and normal zinc-fed mice were observed. Plasma aminotransferase activity was used to evaluate hepatic injury. The mean plasma AST and ALT were significantly elevated in HFD-fed mice compared with CD-fed mice. In HFD-fed mice, zinc supplementation resulted in slightly decreased AST and ALT levels compared with mice fed normal zinc, although the results were not statistically significant. In CD-fed mice, AST and ALT levels were unaffected by dietary zinc supplementation. (Figure 5C,D). Ultimately, the increases in liver injury markers and the progression of NAFLD induced by a HFD were unable to be ameliorated by zinc supplementation.

**Figure 6:**
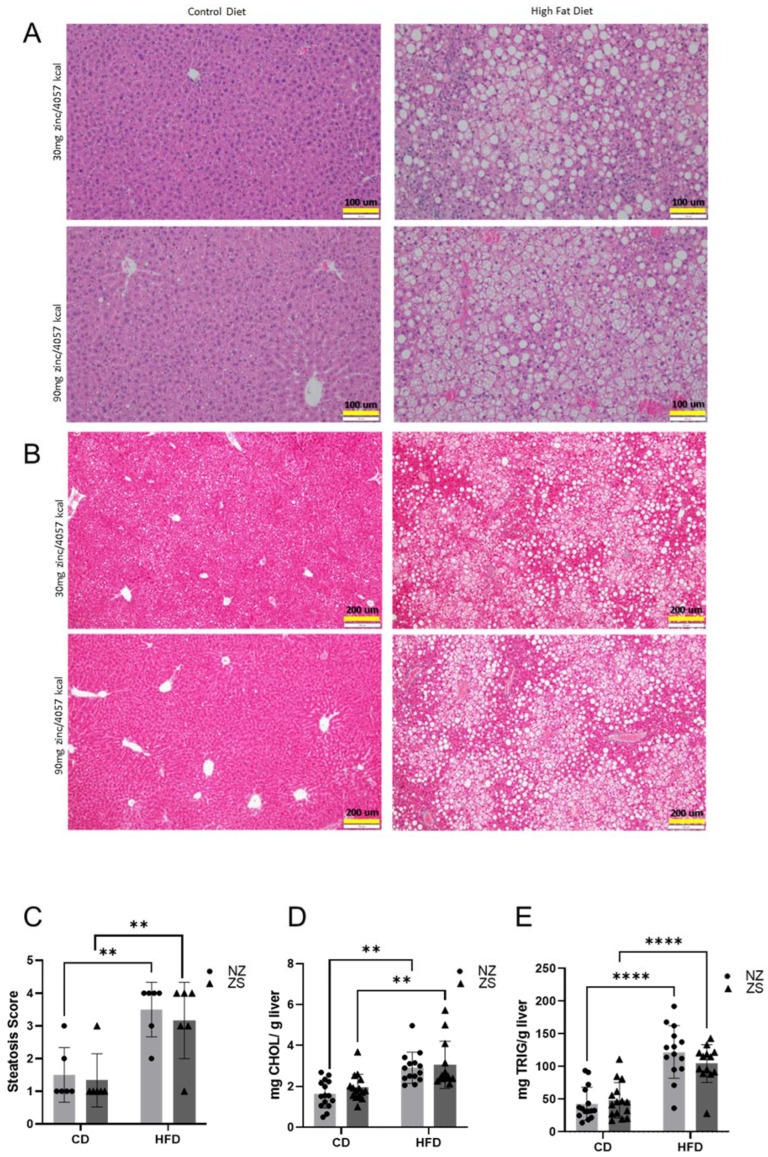
**Effects of HFD and zinc supplementation on hepatic steatosis.** Zinc supplementation did not attenuate HFD-induced steatosis nor increase hepatic triglycerides or cholesterol. 5 μm liver tissue sections stained by (**A**) H&E (20×) and (**B**) Masson Trichrome (10×) showed hepatic morphology and macrovascular steatosis or collagen deposition, respectively. (**C**) Steatosis was scored as percentage of liver cells in 10, 10× field per liver containing fat. (**D**) Hepatic levels of cholesterol (CHOL) and (**E**) triglycerides (TRIG). Data are reported as mean ± SD (n = 6 mice/group, C; n = 14–15 mice/group, **D**,**E**) with significance set to 0.05. ** *p* < 0.05, **** *p* < 0.0001. CD = control diet, HFD = high fat diet, NZ = normal zinc, ZS = zinc supplement.

Histological analysis showed significant increases in steatosis in the HFD-fed mice compared with the CD-fed mice, as seen in representative photomicrographs of H&E (Figure 6A) as well as in the quantification of the histology (Figure 6C). Furthermore, the biochemical analysis of liver tissue revealed mice fed the HFD had greater total cholesterol and hepatic triglyceride levels compared with mice fed the CD (Figure 6D,E). Dietary zinc supplementation did not significantly alter the histological or biochemical parameters of NAFLD in either diet group. No qualitative differences in collagen deposition, as seen in representative photomicrographs of Masson Trichrome, were observed between any of the groups (Figure 6B), and this was confirmed quantitatively (data not shown). Taken together, the data showed HFD was able to induce liver injury, of which zinc supplementation was unable to attenuate.

### 3.6. Altered Gene Expression

**Figure 7:**
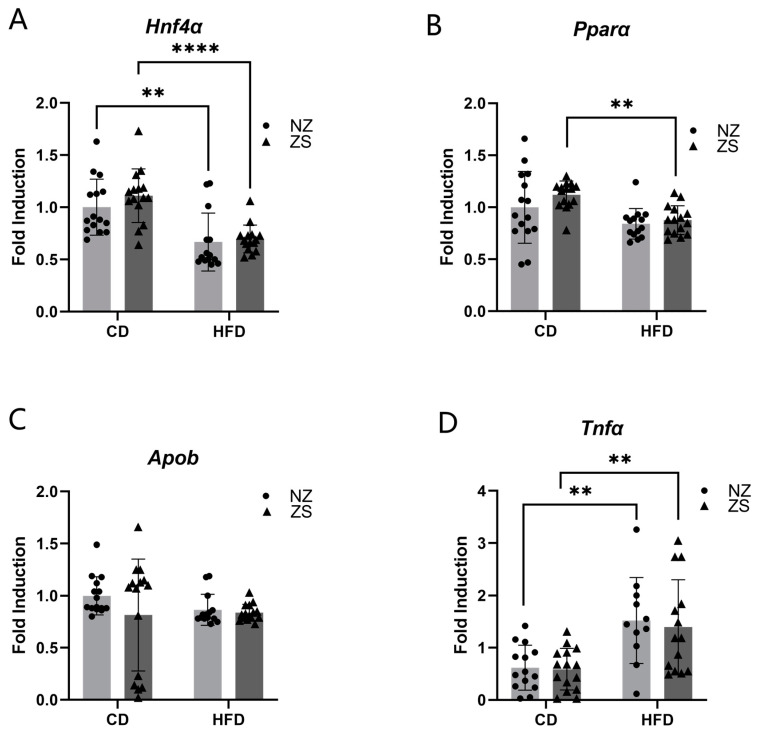
**Effects of zinc on hepatic gene expression.** Zinc supplementation did not affect HFD-induced dysregulated gene expression of transcription factors involved in hepatic function and lipid metabolism. Hepatic (**A**) Hnf4α (**B**) Pparα (**C**) Apob and (**D**) Tnfα mRNA expression. Data are reported as mean ± SD (n = 14–15 mice/group) with significance set to 0.05. ** *p* < 0.05, **** *p* < 0.0001 CD = control diet, HFD = high fat diet, NZ = normal zinc, ZS = zinc supplement. Hnf4α, Hepatocyte nuclear factor 4 alpha; Pparα, Peroxisome proliferator-activated receptor alpha; Apob, apolipoprotein b; Tnfα, Tumor necrosis factor.

Key regulators involved in hepatic function, fatty acid transport, and metabolism were examined by qRT-PCR for changes in gene expression. Expression of *Hnf4α*, a gene responsible for the transcriptional regulation of hepatic progenitor genes, was reduced in HFD-fed mice compared with mice fed the CD (Figure 7A). Independent of fat content, there was a trend toward increased *Hnf4α* expression in zinc-supplemented mice, although they were not significantly different from mice fed normal zinc diets. Similarly, the expression of *Pparα,* a gene involved in the transcriptional regulation of hepatic lipid metabolism, was reduced in HFD-fed mice compared with CD mice (Figure 7B). In CD-fed mice, a non-significant trend of increased *Pparα* expression was observed in the mice supplemented with zinc compared with mice fed a normal zinc diet. In HFD-fed mice, zinc supplementation did not significantly alter *Pparα* expression. Expression of *Apob*, a gene involved in lipid transport, was reduced in zinc-supplemented CD-fed, normal zinc HFD, and zinc-supplemented HFD-fed mice compared with normal zinc CD mice, although there was no statistical difference between the groups (Figure 7C). Increased expression of *Tnfα*, a proinflammatory gene, was observed in HFD-fed mice compared with CD-fed mice (Figure 7D). However, zinc supplementation did not change expression levels of *Tnfα* in either CD- or HFD-fed mice. Overall, zinc supplementation did not alter the expression levels of these selected genes.

## 4. Discussion

Treatment options for NAFLD are limited to dietary and lifestyle changes focused on weight loss and the reversal of syndrome factors. These lifestyle interventions are at times conducted with pharmacological therapies; however, there remains a need for an approved therapeutic agent for the treatment or prevention of NAFLD. Previous studies have shown zinc deficiency is associated with increased metabolic disorders, dyslipidemia, oxidative stress, and inflammation [11,19]. Using zinc as a preventive therapy, dietary zinc supplementation has been shown to diminish alcohol-induced steatosis [21] and improve liver function [27]. In this present study, we developed an in vivo model to test the hypothesis that dietary zinc supplementation can act as a treatment therapy to attenuate established HFD-induced NAFLD. To our knowledge, this is the first animal study investigating the effects of zinc supplementation after NAFLD progression. We successfully induced marked hepatic lipid accumulation by feeding mice a HFD (60% fat-kcal). The results obtained from this study demonstrated that 8-week dietary zinc supplementation after the establishment of fatty liver disease in mice did not significantly lessen or rescue HFD-induced NAFLD. 

While zinc supplementation has been shown to improve lipid and glucose metabolism, which is dysregulated in fatty liver disease progression [21], dietary zinc supplementation from our study was unable to alleviate the disrupted metabolic endpoints associated with HFD-induced NAFLD. Our data is contrary to other studies [10,22] that reported zinc supplementation improved HFD-induced liver injury and decreased hepatic lipid accumulation and serum lipids. In the animal study by Shidfar et al. (2018) [10], rats were fed a HFD for 28 weeks, with zinc and selenium supplementation introduced in the last 8 weeks of the study. Consistent with our results, total cholesterol, HDL, ALT, AST, and glucose, as well as increased levels of steatosis, were elevated in the HFD-fed group. In contrast to our findings, serum triglycerides, cholesterol, ALT, AST, and hepatic steatosis were decreased, while hepatic levels of zinc were increased in zinc-supplemented rats. These differences may be attributed to their use of a rat animal model as opposed to the murine model used in our study. Additionally, the supplementation after disease progression was composed of both zinc and selenium, versus our investigation of the effects of zinc supplementation alone. Their results indicate the potential necessity of an additional micronutrient in addition to zinc to provide synergistic therapeutic effects on adverse phenotypes of NAFLD, or the positive effects of the combined supplementation may be limited to the rat animal model.

More comparable to our study is the murine HFD model study by Qi et al. (2020) [22]. Results from their study correlated with our observations of increased obesity, hepatic lipid accumulation, and liver injury in HFD-fed mice compared with control diet mice. However, while dietary zinc supplementation did not alter metabolic endpoints in our study, Qi et al. (2020) reported improvements in fat and lean mass, glucose tolerance, hepatic injury, and lipid deposition in zinc-supplemented HFD-fed mice [22]. Possible explanations for these paradoxical differences in results may be that, whereas in our study zinc supplementation was introduced in the last 8 weeks after disease progression, Qi et al. (2020) began administering zinc supplementation concurrently with HFD for a total period of 14 weeks [22]. This suggests there may be a certain threshold of NAFLD progression beyond which zinc supplementation is unable to have a therapeutic effect. Supplementation may be required well in advance of the establishment of NAFLD to have the desired restorative benefits. The implication of this is that patients begin taking recommended dietary zinc supplementation either ahead of or with the consumption of a high-fat diet, of which the likelihood may be rather low.

Pparα and Hnf4α are zinc finger transcription factors that play key regulatory roles in hepatic gene expression, lipid homeostasis, and very-low-density lipoprotein (vLDL) secretion [28,29]. Hnf4α affects hepatic fat storage through the induction of lipophagy, while Pparα, a nutritional sensor, is essential to lipid transport and β-oxidative pathways. Our data showed decreased mRNA expression of Pparα and Hnf4α in HFD-fed mice. This is consistent with literature showing Hnf4α activity is decreased by fatty acids and decreased Pparα results in enhanced steatosis. In the alcohol-induced model of steatosis described by Kang et al. (2009) [21], zinc supplementation was reported to significantly increase the expression levels of these genes, which were unaffected by alcohol. Our results similarly showed slight increases in Pparα and Hnf4α expression due to zinc supplementation.

While our data did not confirm our hypothesis of zinc supplementation attenuating established NAFLD, we have not looked at other parameters that may have contributed to the null findings. Zinc is primarily absorbed into circulation through the gut [13], and gut permeability and the microbiome are altered in NAFLD [30]. How HFD may have altered gut permeability before and after zinc supplementation in this model is yet to be investigated. Studies on this may elucidate changes to zinc transporters in the gut that may have resulted in unchanged hepatic zinc levels between zinc-supplemented and normal zinc mice.

Potential limitations of this study may include the fat percentage of the diet administered to the HFD group. The 60% fat diet is known to cause rapid obesity in mice but presents a much higher distortion of the fat content compared with the normal rodent chow, resulting in exaggerated metabolic responses [31]. The rapid induction of obesity may have also accelerated the development of NAFLD, exceeding attenuation by zinc supplementation. While a 60% fat-kcal HFD was used in the study by Qi et al. (2020), it should again be noted that the introduction of the diet and zinc supplementation began at the same time [22]. In future studies, it may be more beneficial to utilize the 45% fat rodent diet, as obesity in mice can be achieved with the 45% fat diet, albeit more slowly, and this may be more relevant to human physiology than the 60% fat diet. Additionally, the period of zinc supplementation may have been too brief for a striking phenotypic difference to be detected between normal zinc and zinc-supplemented mice. Qi et al. (2020) provided zinc supplementation for a total duration of 14 weeks [22]. The longer treatment period may be a necessary requirement for the desired therapeutic effects of zinc on the liver. Lastly, zinc deficiency plays a vital role not only in disease progression but also in the efficacy of zinc supplementation in the reversal of excess hepatic lipids. In alcohol-associated liver disease, micronutrient depletion is commonly noted and rectified by replenishing essential micronutrients. Particularly, clinical studies have established significantly low serum and liver zinc concentrations in patients with alcohol-induced steatosis, hepatitis, and cirrhosis [32]. Our study did not assess zinc status prior to introducing zinc supplementation, which would impact the effectiveness of the supplemented zinc. On the one hand, if our HFD-fed mice were zinc deficient after 12 weeks, improved hepatic lipids and glucose metabolism may have been observed after zinc supplementation. On the other hand, if our HFD-fed mice were not zinc deficient after 12 weeks, the additional zinc may have been simply excreted out of the body, explaining why there were no observed differences in hepatic zinc levels. These limitations will be addressed in our future studies.

Taken together, our data demonstrate the use of zinc supplementation as an effective therapeutic for the treatment of established NAFLD, which will require a sensitive consideration of the initiation and duration of administration for developed NAFLD. Further research insights are required to elucidate and ascertain underlying mechanisms to provide desirable outcomes of zinc supplementation for NAFLD.

## Figures and Tables

**Table 1:** Plasma levels of total cholesterol, triglycerides, HDLs, LDLs, vLDLs.

	CD-NZ (n)	CD-ZS (n)	HFD-NZ (n)	HFD-ZS (n)
**Cholesterol**	109.8 ± 7.5 (14)	110.7 ± 4.5 (15)	178.4 ± 10.4 (14) #	163.5 ± 9.5 (14) #
**Triglycerides**	73.2 ± 5.6 (14)	74.7 ± 5.0 (15)	58.4 ± 5.3 (14)	54.1 ± 4.1 (14) #
**HDL**	84.4 ± 6.2 (14)	85.3 ± 3.2 (15)	96.9 ± 2.5 (14)	97.1 ± 2.0 (14)
**LDL**	8.8 ± 1.3 (12)	8.3 ± 1.5 (13)	14.0 ± 10.0 (2)	7.0 ± 1.0 (2)
**vLDL**	14.7 ± 1.1 (14)	15.1 ± 1.0 (15)	11.6 ± 1.1 (14)	10.8 ± 0.8 (14) #

Data reported as mean ±S.E.M. (mg/dL), with significance set to 0.05. # *p* < 0.05 compared with mice fed normal zinc or zinc supplemented control diet. CD= control diet, HFD = high-fat diet, NZ = normal zinc, ZS = zinc supplement.

## Data Availability

Not applicable.
